# Osteoprotegerin Inhibits Calcification of Vascular Smooth Muscle Cell via Down Regulation of the Notch1-RBP-Jκ/Msx2 Signaling Pathway

**DOI:** 10.1371/journal.pone.0068987

**Published:** 2013-07-09

**Authors:** Shaoqiong Zhou, Xing Fang, Huaping Xin, Wei Li, Hongyu Qiu, Siming Guan

**Affiliations:** 1 Department of Gerontology, Union Hospital, Tongji Medical College, Huazhong University of Science and Technology, Wuhan, China; 2 Department of Cell Biology and Molecular Medicine, New Jersey Medical School, University of Medicine and Dentistry of New Jersey (UMDNJ), Newark, New Jersey, United States of America

## Abstract

**Objective:**

Vascular calcification is a common pathobiological process which occurs among the elder population and in patients with diabetes and chronic kidney disease. Osteoprotegerin, a secreted glycoprotein that regulates bone mass, has recently emerged as an important regulator of the development of vascular calcification. However, the mechanism is not fully understood. The purpose of this study is to explore novel signaling mechanisms of osteoprotegerin in the osteoblastic differentiation in rat aortic vascular smooth muscle cells (VSMCs).

**Methods and Results:**

VSMCs were isolated from thoracic aorta of Sprague Dawley rats. Osteoblastic differentiation of VSMCs was induced by an osteogenic medium. We confirmed by Von Kossa staining and direct cellular calcium measurement that mineralization was significantly increased in VSMCs cultured in osteogenic medium; consistent with an enhanced alkaline phosphatase activity. This osteoblastic differentiation in VSMCs was significantly reduced by the addition of osteoprotegerin in a dose responsive manner. Moreover, we identified, by real-time qPCR and western blotting, that expression of Notch1 and RBP-Jκ were significantly up-regulated in VSMCs cultured in osteogenic medium at both the mRNA and protein levels, these effects were dose-dependently abolished by the treatment of osteoprotegerin. Furthermore, we identified that Msx2, a downstream target of the Notch1/RBP-Jκ signaling, was markedly down-regulated by the treatment of osteoprotegerin.

**Conclusion:**

Osteoprotegerin inhibits vascular calcification through the down regulation of the Notch1-RBP-Jκ signaling pathway.

## Introduction

Vascular calcification is a common pathological process [1] which is observed extensively in patients with renal disease [2] and type II diabetes [3] as well as in aging populations [4–6]. Although the clinical significance of vascular calcification is well-recognized [6], the mechanisms involved are still not clear. It is accepted that plasticity and phenotypic modulation of vascular smooth muscle cells play a pivotal role in the development of vasculature calcification [5,7,8].

Osteoprotegerin (OPG), also known as osteoclastogenesis inhibitory factor [OCIF] or the tumor necrosis factor (TNF) receptor-like molecule[TR1], a member of the TNF family of receptors, is a secreted protein that regulates bone mass by inhibiting osteoclast differentiation and activation [9]. OPG-deficient mice exhibited a decrease in total bone density as well as a high incidence of fractures [9,10], and also developed calcified lesions in the aorta and renal arteries [9,11]. However, the vascular calcification would be negligent in this mouse if recombinant OPG protein was supplied with in the embryonic period. These results suggest that OPG acts as an important inhibitor of the development of vascular calcification. However, the molecular signaling pathway of OPG mediated vascular calcification is not completely understood.

Notch is a family of transmembrane proteins that is only found in Metazoans or animals. Notch exists at the cell surface in heterodimeric form (cleaved by furin in the rans-Golgi) or as an intact (colinear) protein. 4 mammalian Notch receptors (Notch-1 to Notch-4) corresponding with at least five ligands termed Jagged 1, Jagged 2, Delta -1, Delta -2, Delta -3 were found [12]. The interaction of Notch with a ligand results in the extracellular processing of the Notch receptor by a disintegrin-metalloprotease that releases the intracellular domain of Notch to the nucleus and facilitates an association with the transcription factor RBP-Jκ (also known as CBF-1 or CSL). The subsequent recruitment of the coactivator, mastermind-like (MAML) protein, promotes transcriptional activation of downstream effectors [13]. It is shown that Notch receptors play a role in controlling the human VSMC phenotype and repressing VSMC differentiation in a RBP-Jκ-dependent manner in vitro [12,14]. Recent studies showed that Notch1-RBP-Jκ signaling pathway is involved both in phenotypic modulation of VSMCs and osteo/chondrogenesis [15].

Therefore, in the present study, we performed an in vitro study to test our hypothesis that OPG inhibits the development of vascular calcification by inhibiting the Notch1-RBP-Jκ signaling pathway. By using Von Kossa staining, we found that that OPG markedly inhibits the osteoblastic differentiation of VSMCs which is further confirmed by the Alkaline Phosphatase (ALP) Assay and calcium content measurement. Furthermore, we identified that OPG significantly down regulated the Notch1-RBP-Jκ signaling pathway and its downstream target MSX2.

## Materials and Methods

### 1: Cell Culture and treatments

Primary vascular smooth muscle cells (VSMCs) were isolated from the descending thoracic aorta of Sprague Dawley (SD) rats (4 weeks old, male) as described previously [16]. The protocol was approved by the Institute Animal Care and Use committee of Tongji Medical College, Huazhong University of Science and Technology, Wuhan, China (Permit Number: SYXY2010-0057). The study was carried out in strict accordance with the recommendations in the Guide for the Care and Use of Laboratory Animals of the Experimental Animals Management Committee (Hubei Province, China). All animals were sacrificed under anesthesia to minimize suffering. Briefly, rats were sacrificed under anesthesia with intraperitoneal injection of sodium pentobarbital (120 mg/kg). The thoracic aorta was isolated and washed in PBS three times. All external fat and connective tissue were detached and the adventitia and endothelial layer were carefully removed. Strips of media were incubated with Dulbecco’s modified Eagle’s medium (DMEM) with 20% FBS (Cell applications, Inc.). Confirmation of the phenotype was obtained by fluorescent immunostaining for α-smooth muscle actin. Cells were maintained in Dulbecco’s modified Eagle’s medium (DMEM) with 10% FBS, 100U/ml penicillin, and 100 mg/ml streptomycin and were incubated in 75 cm^2^ tissue culture flasks at a density of 1×10^4^ cells/ml. VSMCs at passage 5 to 6 were used for the experiments of this study.

Osteoblastic differentiation was induced by culturing cells in an osteogenic medium, containing 50 mg/ml ascorbate-2-phosphate and 10mM β-glycerol phosphate. DMEM was used as culture control. N-[N-(3,5-Difluorophenacetyl) -L-alanyl]-S-phenylglycinet-butyl ester (DAPT) (10mM), a known potent inhibitor of the Notch1-RBP-Jκ dependent signaling pathway, was used as a positive control. VSMCs were also cultured in the presence of different concentrations of OPG (0.1, 1, and 10ng/ml). After 14 day treatments, cultured VSMCs were submitted to the following experiments. A total of six groups were included in this study: control (VSMC cultured with DMEM); osteogenic (OS) group (VSMC cultured with osteogenic medium only); DAPT group (VSMC cultured with OS and DAPT); OPG groups (VSMC cultured with OS and OPG), which are divided into three subgroups due to the different concentration of OPG (0.1, 1, and 10ng/ml).

### 2: Von Kossa Staining

Calcium deposition in cultured VSMCs was investigated using Von Kossa staining as previously described [17]. VSMCs in culture flasks were washed 3 times with PBS, followed by a fixation with 4% paraformalclehyde for 15 min. The cells were then washed 3 times with distilled water and incubated with 5% silver nitrate solution and exposed to bright sunlight for 30 min, then washed with distilled water for 5min, and treated with 5% sodium thiosulfate for 2min. Calcium particles were observed in visual fields at a magnification of ×40.

### 3: Measurement of intracellular calcium content

The cultures were decalcified with 0.6 N HCl for 24 h. After decalcification, the cells were washed with PBS and solubilized with 0.1 N NaOH-0.1% SDS. The calcium content of the HCl supernatant was determined by the ocresolphthalein Complexone method (calcium kit, NanJingJian-Cheng Bioengineering Institute). The cell number is normalized by protein amount of VSMCs.

### 4: Alkaline Phosphatase (ALP) Assay

ALP activity of VSMCs was measured using Lab Assay ALP (kit) (NanJing-JianCheng Bioengineering Institute), according to the manufacturer’s protocol. Briefly, cultured cells are washed with PBS and lysed in 0.5 mL 0.2% Triton X-100 in distilled water by shaking for 20 min at room temperature. p-nitrophenyl phosphate that is hydrolyzed by ALP into a yellow colored product was detected (maximal absorbance at 405nm). The rate of the reaction was directly proportional to the enzyme activity. The cell number was normalized by protein amount of VSMCs.

### 5: Real-time quantitative PCR

Total RNA was isolated from the cultured VSMCs using Trizol chloroform method reagent according to the manufacturer’s instructions (Invitrogen, USA) and reverse transcribed into cDNA with a Toyoba reverse transcription kit (Fermentas, Canada). The real-time quantitative PCR was carried out with the ABI PRISM 7900 sequence detector system (Applied Biosystems, Foster City, Canada) according to the manufacturer’s instructions. The β-actin was used as endogenous control. PCR reaction mixture contained the SYBR Green/Fluorescein QPCR Master Mix (2X) (Fermentas, Canada), cDNA, and the primers. Relative gene expression level (the amount of target, normalized to endogenous control gene) was calculated using the comparative Ct method formula 2^-ΔΔCt^. The sequences of primers for real-time quantitative PCR were:

Rat Notch 1 152bp
Rat Notch1 Forward: 5’- GAGGCTTGAGATGCTCCCAG -3’
Rat Notch1 Reverse: 5’- ATTCTTACATGGTGTGCTGAGG -3’
Rat msx2 163bp
Rat msx2 Forward: 5’- AAGGCAAAAAGACTGCAGGA -3’
Rat msx2 Reverse: 5’- GGATGGGAAGCACAGGTCTA -3’
Rat RBP-Jκ 232bp
Rat RBP-Jκ Forward: 5’- GAGCCATTCTCAGAGCCAAC -3’
Rat RBP-Jκ Reverse: 5’- TCCCCAAGAAACCACAAAAG -3’
β-actin 240bp
β-actin Forward: 5’- CACGATGGAGGGGCCGGACTCATC-3’
β-actin Reverse: 5’- TAAAGACCTCTATGCCAACACAGT -3’

### 6: Western blotting analysis

Protein was extracted from cultured VSMCs in radio immunoprecipitation assay buffer (RIPA), containing 50mM Tris, 150 mM NaCl, 0.1% SDS, 0.5% sodium deoxycholate, 1% Triton X-100 in the presence of aprotinin, PMSF, okadaic acid, and leupeptin. Total protein (50 µg) per sample was loaded onto 12% SDS-polyacrylamide gels and separated at 100 V followed by transfer to PVDF at 200mA. Membranes were blocked in 5% non-fat milk in 0.1M PBS, pH 7.4 at room temperature for 2h and then incubated with primary antibodies: goat anti-Notch1 polyclonal antibody (1:400) (Santa, USA), or rabbit anti-RBP-Jκ polyclonal antibody (1:300) (Santa, USA) or goat anti-Msx2 polyclonal antibody (1:400) (Santa, USA). After washing, membranes were incubated in HRP conjugated rabbit-anti-goat or goat-anti-rabbit secondary antibody (1:40000) for 2 hours at room temperature followed by washing and 5 min incubation with ECL reagents. The membranes were stripped and equal protein loading was determined by GAPDH expression using a mouse monoclonal antibody (1:75,000).

### 7: Statistical analysis

The results are shown as mean± SE. The significance of differences was estimated by ANOVA followed by Student-Newmann-Keuls multiple comparison tests. P≤0.05 was considered statistically significant. All statistical analyses were performed using SPSS soft-ware (version 17.0, SPSS Inc., Chicago, IL).

## Results

### 1: OPG inhibits osteoblastic differentiation of VSMCs

**Figure 1:**
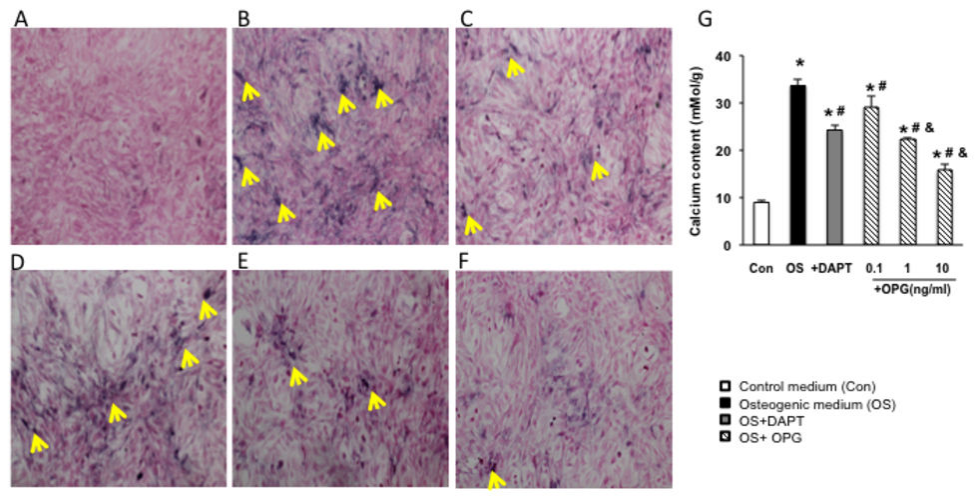
VSMC calcification. A to F Von Kossa staining of VSMCs. Cells were culture in different medium for 14 days. (A) Von Kossa staining of VSMCs cultured in control DMEM medium. (B) Von Kossa staining of VSMCs cultured in osteogenic medium. (C) Von Kossa staining of VSMCs treated with DAPT. (D) -(F) Von Kossa staining of VSMCs treated with different concentrations of OPG: 0.1ng/ml(d), 1ng/ml(e) and 10ng/ml(f) (arrows showed the positive stained calcium particles, black color). G. Cellular calcium content of VSMCs. Calcium content in VSMCs in osteogenic medium was increased by 5 folds in the VSMCs cultured with compared to the control; OPG reduced the calcium deposition in VSMCs induced by osteogenic stimulation by 20 to 60% in a dose dependent manner (p<0.05, compared to VSMCs cultured in osteogenic medium). These observations together indicate the OPG plays an important role in the regulation of the osteoblastic differentiation of VSMC. *, P<0.05 vs control, #, P<0.05 vs osteogenic medium, and &, P<0.05 vs OPG 0.1ng/ml. n=3.

To determine the effect of OPG on osteoblastic differentiation of VSMCs, Von Kossa staining was used to detect calcium deposition in VSMCs. As showed in Figure 1A, calcium particles were not detected in VSMCs cultured in control DMEM medium, while positively-staining particles (black as showed by arrows) were significantly increased in VSMCs cultured in osteogenic medium (Figure 1B), indicating an increase of osteoblastic differentiation in these VSMCs. The increase of osteoblastic differentiation was significantly reduced by the treatment of DAPT, a gamma secretase inhibitor that abrogates Notch signaling by interrupting cleavage of Notch intracellular domain upon ligand stimulation, (Figure 1C). Importantly, this enhanced calcium deposition was also markedly reduced in the presence of OPG in a dose-dependent manner (Figure 1D to 1F) compared with VSMCs cultured in osteogenic medium only (Figure 1B). This indicates that OPG, similar to DAPT, repressed the osteoblastic differentiation of VSMCs induced by osteogenic media (Figure 1). These observations were further confirmed by the quantitative measurement of the cellular calcium content. As shown in Figure 1G, the cellular calcium content was increased by 5 fold in VSMCs cultured in osteogenic medium compared to the control (p<0.05). We also showed that OPG reduced the calcium deposition in VSMCs induced by osteogenic stimulation by 20 to 60% in a dose dependent manner (p<0.05, compared to VSMCs cultured in osteogenic medium). These observations together indicate the OPG plays an important role in the regulation of the osteoblastic differentiation of VSMC.

### 2: ALP Assay in VSMCs treated with OPG

**Figure 2:**
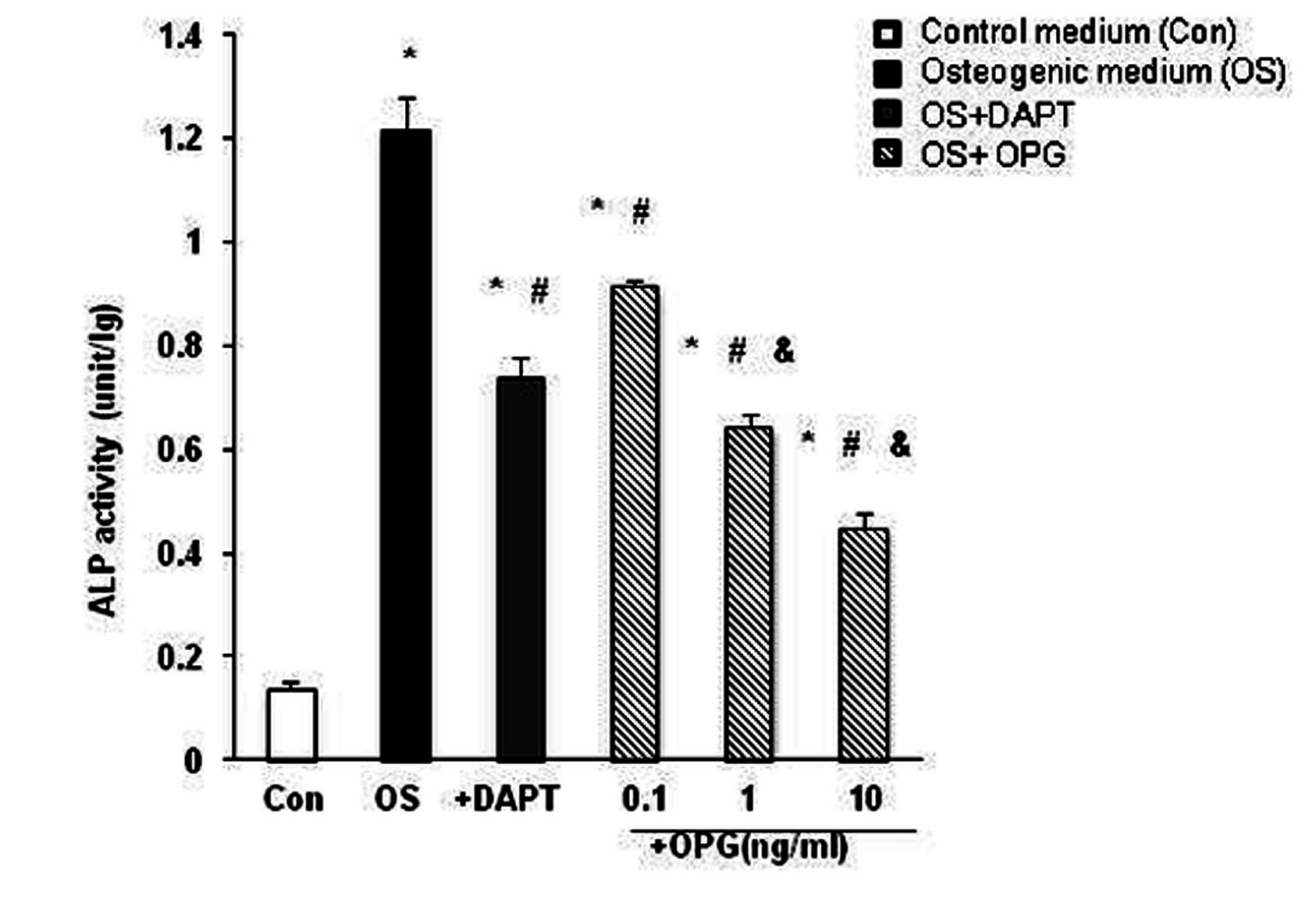
Alkaline Phosphatase activity of VSMCs. The ALP activity was measured and normalized by protein amount in VSMC from different media. ALP activity was significantly increased by 3.5 folds in VSMCs treated with osteogenic media compared to the control, confirming a osteogenic conversion in the VSMCs. The enhanced ALP activity induced by osteogenic medium was repressed significantly by 30% with the addition of DAPT and also significantly reduced by OPG dose dependently. *, P<0.05 vs control, #, P<0.05 vs osteogenic medium, and &, P<0.05 vs OPG 0.1ng/ml. n=3.

ALP activity, an early marker of osteogenic conversion, was determined in cultured VSMCs. As shown in Figure 2, ALP activity was significantly increased by 3.5 fold in VSMCs in osteogenic media compared to the control (p<0.05), confirming an osteogenic conversion in the VSMCs. The enhanced ALP activity induced by osteogenic medium was repressed significantly by 30% with the addition of DAPT and also significantly reduced by OPG dose dependently (p<0.05 versus the osteogenic VSMCs). These results are consistent with the reduced calcium deposition observed in VSMCs in the presence of OPG presented in Figure 1, which further confirms the inhibitive effects of OPG on the osteogenic conversion of VSMCs.

### 3: OPG down regulates the Notch1-RBP-Jκ-dependent signaling pathway

**Figure 3:**
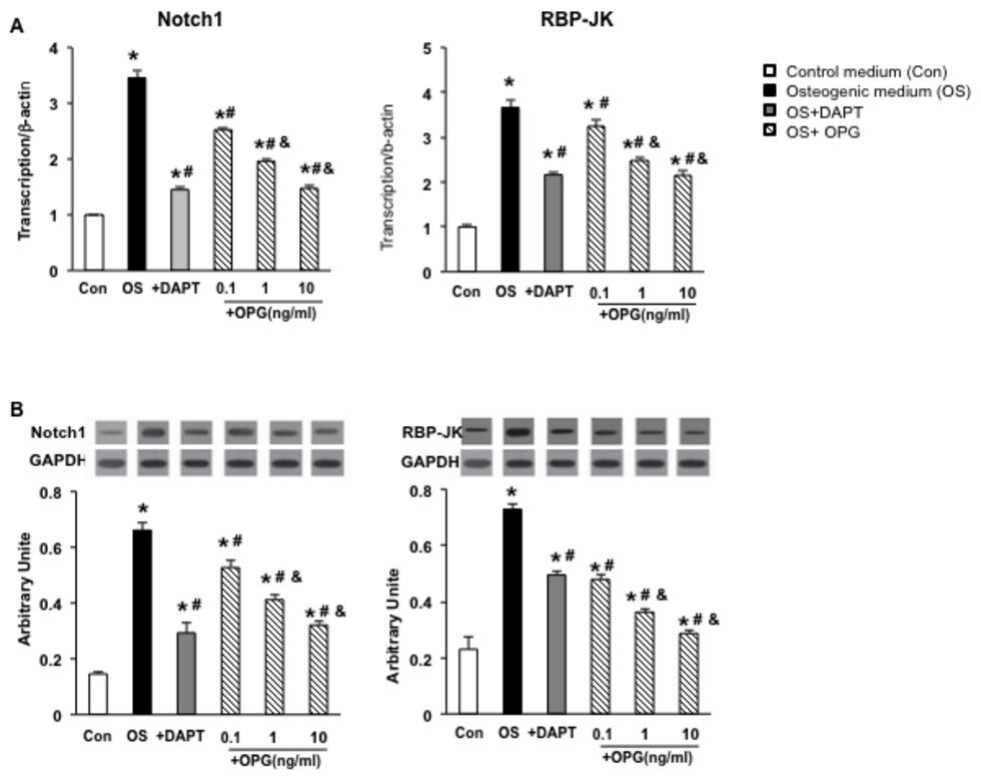
Expression of Notch1 and RBP-Jκ in VSMCs. (A) mRNA level of Notch1 and RBP-Jκ were measured by real-time RT-PCR in VSMCs cultured in different media. (B) Protein level of Notch1 and RBP-Jκ were measured by Western blotting in VSMCs cultured in different media. Notch1 and RBP-Jκ significantly increased by 3 to 4 folds in VSMC cultured in osteogenic medium compared to control at both mRNA and protein levels, suggesting that the Notch1-RBP-Jκ signaling pathway is activated in osteogenic conversion of VSMCs. DAPT reduced the expression of Notch1 by 50% and RBP-Jκ by 30% in VSMCs compared to osteogenic cells at bothe mRNA and protein levels. Similarly, OPG reduced the expression of Notch1 and RBP-Jκ compared to VSMCs in the osteogenic medium in a dose dependent manner. *, P<0.05 vs control, #, P<0.05 vs osteogenic medium, and &, P<0.05 vs OPG 0.1ng/ml. n=3.

Rat aortic VSMCs were treated with different media as described in the Methods. mRNA and protein levels of Notch1 and RBP-Jκ in VSMC were measured by real-time RT-PCR and Western blotting. As shown in Figure 3, Notch1 and RBP-Jκ were significantly increased by 3 to 4 fold in VSMCs cultured in osteogenic medium compared to the control at both the mRNA and protein levels (*P<0.05 vs control), suggesting that the Notch1-RBP-Jκ signaling pathway is activated in the osteogenic conversion of VSMCs (Figure 3).

To determine the effects of OPG on the expression of the Notch1-RBP-Jκ dependent signaling pathway in VSMCs, three different concentrations (0.1ng/l, 1ng/l and 10ng/l) of OPG was added to VSMCs cultured in osteogenic media, and the DAPT was added as a positive control of inhibition of the Notch1--RBP-Jκ-dependent signaling pathway. Our data showed that DAPT reduced the expression of Notch1 by 50% and RBP-Jκ by 30% in VSMCs compared to osteogenic cells at both the mRNA and protein levels. Similarly, OPG reduced the expression of Notch1 and RBP-Jκ compared to VSMCs in osteogenic medium in a dose dependent manner (#. P<0.05, compared to osteogenic VSMCs (Os group)), indicating its inhibitive effect on the Notch1--RBP-Jκ-signaling pathway.

### 4: OPG represses downstream target of Notch1-RBP-Jκ-signaling pathway in VSMCs

**Figure 4:**
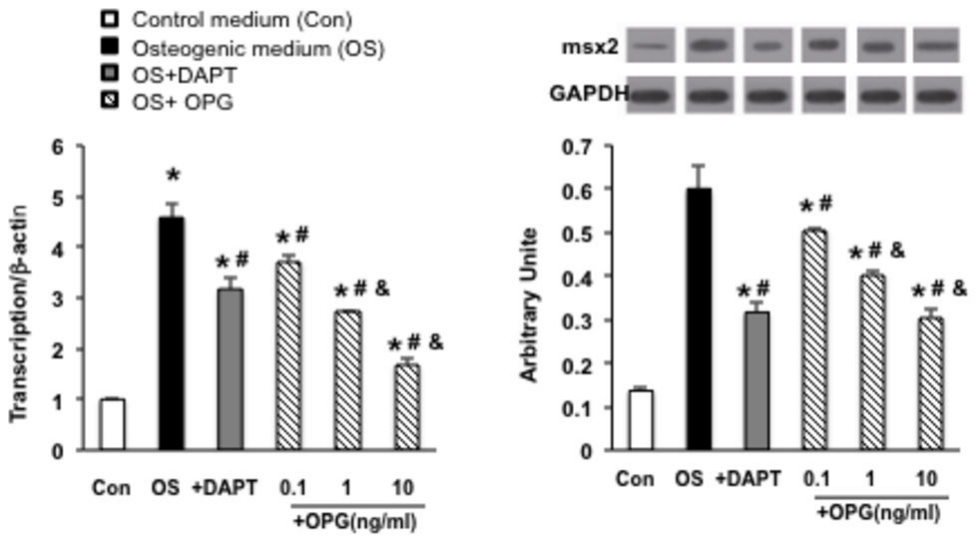
Expression of Msx2 in VSMCs. (A) mRNA level of msx2 was measured by real-time RT-PCR in VSMCs cultured in different media in VSMCs cultured in different media. (B) Protein level of Msx2 was measured by Western blotting in VSMCs cultured in different media. The expression of Msx2 was significantly increased with the treatment of osteogenic medium by 3 to 4 fold compared with control at both mRNA and protein levels. The activation of Msx2 by osteogenic media was deteriorated by the addition of DAPT; the addition of OPG significantly reduced the expression of Msx2 in a dose dependent manner, indicating that OPG represses expression of Msx2 through the reduction of the Notch1-RBP-Jκ-signaling pathway. *, P<0.05 vs control, #, P<0.05 vs osteogenic medium, and &, P<0.05 vs OPG 0.1ng/ml. n=3.

Next, we tested whether inhibition of the Notch1-RBP-Jκ­signaling pathway affected the downstream target gene Msx2. As showed in Figure 4, the expression of Msx2 was significantly increased in osteogenic medium by 3 to 4 fold compared with the control at both mRNA and protein levels (*P<0.05), which corresponded with an increase of Notch1 and RBP-Jκ. To test whether up-regulation of Msx2 by osteogenic media is Notch1-RBP-Jκ­dependent, DAPT was added. The results in Figure 4 show that the activation of Msx2 by osteogenic media was significantly diminished by the addition of DAPT (P<0.05 compared to VSMCs cultured in osteogenic media), which is consistent with the inhibition of the expression of Notch1-RBP-Jκ (Figure 3). This further confirms that the osteoblastic differentiation of VSMCs induced by osteogenic medium is mediated by Notch1-RBP-Jκ signaling. Importantly, we observed that addition of OPG significantly reduced the expression of Msx2 in a dose dependent manner, indicating that OPG represses expression of Msx2 through the suppression of the Notch1-RBP-Jκ-signaling pathway.

## Discussion

Despite the fact that OPG has been proven to participate in multiple aspects of vascular calcification [15,17], the molecular mechanism(s) underlying its function remains exclusive. Whereas previous work has focused on the investigation of OPG on the osteoclastogensis in osteoclast development, our current study focused on the regulation of OPG on osteoblastic differentiation of VSMCs and the signaling pathway involved in this function. Our results show that not only is OPG an important inhibitor of osteoblastic conversion in VSMCs, but that it also inhibits VSMC calcification by blocking the Notch1-RBP-Jκ-dependent signaling pathway. These findings provide solid evidences implicating OPG as a key regulator of rat aortic calcification.

Osteoprotegerin (OPG) is a soluble glycoprotein which belongs to a member of tumor necrosis factor (TNF) receptor super family which was initially found in bone [18], but is also found in various other tissues like arterial wall [19,20]. OPG has been linked to diabetes mellitus, silent myocardial ischemia, acute myocardial infarction, and left ventricular dysfunction [21]. The tissue concentration of OPG in aorta and hip-bone is almost identical but 500 times higher than the plasma concentration [22]. OPG exerts its function through binding and neutralizing the receptor activator for nuclear factor kappa B (NF-κB) lignad (RANKL) [23]. In bone, OPG inhibits bone resorption, whereas RANKL promotes bone resorption. Although the function of OPG in arterial wall is not fully known, it has been reported that, contrary to their action in bone, OPG exerts a protective effect on the calcification in the vasculature [24]. This observation was further supported by the study in OPG knockout mouse model, where it has been shown that targeted deletion of OPG in mice results in severe, early-onset osteoporosis, and the calcification of the aorta and renal arteries [10]. OPG was found to inhibit vascular calcification induced by warfarin and vitamin D3 in vivo [25]. These findings suggest that OPG plays an important role in osteoporosis and vascular calcification. Therefore, OPG may act as a potential inhibitor of the development of vascular calcification. In the present study, we focused on aortic vascular smooth muscle cells, a major component of aortic wall, to determine the effect of OPG on the osteogenic conversion of VSMCs. We first identified that osteoblastic differentiation of VSMCs was induced by osteogenic medium which was confirmed by an increase of calcium deposition and ALP activity, a significant marker for osteogenic differentiation, Furthermore, we identified that OPG significantly inhibited the osteogenic conversion of VSMCs in vitro in a dose dependent manner, indicating that repression of osteoblastic differentiation of VSMCs may be a crucial cellular mechanism of the inhibition of vascular calcification by OPG (Figures 1 and 2).

Although experimental evidence indicates that OPG may serve as a vascular calcification inhibitor, emerging clinical observations demonstrated that serum OPG positively correlated with incidence of cardiovascular disease (CVD) and mortality in elders [26–35]. Clinical research also showed that OPG levels are significantly higher in patients with chronic kidney disease (CKD) compared with age- and sex-matched controls, and increasing OPG levels have a linear relationship with adverse renal function [36–39]. Elevated OPG is associated with all-cause mortality in CKD stage 4 and 5 patients in addition to vascular calcification [40]. However, it is not clear whether the elevated serum OPG level is along the causal pathway in the development of these diseases or rather serves as a marker of disease burden. Animal models and experimental data showing that OPG has a rather protective effect against vascular calcification implies that higher OPG levels in patients with CVD and CKD might be a compensatory response to these diseases, rather than a risk factor. Rapid decline in serum OPG levels in these patients after renal and cardiac transplantations [41,42] support the notion that increased serum OPG is a modulatory response to CVD and CKD. With very high concentrations in normal arterial wall, it is likely that the level of OPG in circulating blood reflects arterial content to some degree. It is moreover plausible that increased circulating levels may reflect injury to the arterial wall, putatively as result of the influence of pro-inflammatory molecules on arterial cells [24]. However, there is no direct evidence showing inhibitory effect of OPG in the vascular calcifications. In addition, although it has been shown that OPG knockout mice develop vascular calcification [10], it is unidentified whether vascular calcification displayed in OPG knockout mice is due to the preliminary effects of OPG or due to secondary effects initiated by circulating bone degradation product. Our results in the present study provide direct evidence for the inhibitory effect of OPG on vascular calcification of VSMCs. Importantly, we found that osteoblastic differentiation of VSMCs induced by osteogenic medium was also blocked by DAPT, a specific inhibitor of Notch signaling pathway (Figures 1 and 2), implying that this pathway may be involved in the mechanism of OPG in the regulation of osteoblastic conversion of VSMCs. Therefore our second goal is to determine the effect of OPG on the Notch signaling pathway.

It is known that the Notch signaling pathway plays a key role in the differentiation of many tissues [14,43,44]. It is an evolutionarily conserved pathway that is a critical determinant of the cell fate [45] and adult tissue renewal. Attenuated Notch signaling profoundly enhances osteoclastogenesis and bone resorption in vitro and in vivo by a combination of molecular mechanisms. Recent studies showed that the Notch1 signaling pathway induces osteogenic differentiation and mineralization of VSMCs [14] and inhibiting the Notch1 signaling pathway suppresses calcification of aortic valve [44,46]. These findings combined with our results shown above indicate a potential regulatory function exerted by OPG and the Notch1 signaling pathway in the regulation of osteogenic conversion of VSMCs. Although different Notch signaling pathways were found in various tissues, recent studies showed that Notch-RBP-Jκ MSX2 signaling pathway plays an important role in vascular calcification, not only due to the finding of the colocalization of notch1 and Msx2 in human fibrocalcific plaques, but also due to the direct evidence showing that Notch signaling promotes osteogenic differentiation and mineralization of VSMCs by directly activation Msx2 gene transcription via RBP-Jκ^13^. RBP-Jκ, a major mediator of Notch signaling, binds with notch intercellular domains (NICD) and forms a complex that further activates transcription of target genes from its cognate DNA binding sequence in the nucleus. Msx2, a key osteogenic regulatory factor of vascular calcification, is the downstream target gene of Notch1-RBP-Jκ-dependent signaling pathway [47,48]. It is confirmed that Msx2 mediated Notch1 induced osteogenic conversion of human aortic SMCs via RBP-Jκ^13^. The decrease of Msx2 implies the inhibition of osteogenic differentiation of VSMCs. Our results confirmed that the expression of Notch1 and RBP-Jκ as well as its downstream target Msx2 were significantly increased in osteogenic VSMCs which consist with the increased intracellular calcium deposition. Importantly, the osteogenic effects on VSMCs which include an increase of Msx2, and enhancement of ALP activity as well as an increased deposition of calcium, were significantly reduced by the addition of DAPT, a specific inhibitor that abrogates Notch signaling by interrupting cleavage of Notch intracellular domain upon ligand stimulation. This implies that the Notch1-RBP-Jκ-dependent signaling pathway contributes to the calcification of VSMCs, and that inhibiting the Notch1-RBP-Jκ-dependent signaling pathway represents a potential therapeutic approach for the vascular diseases among the elderly and patients with diabetes and chronic kidney disease. Moreover, our results in this study have identified that OPG inhibits the intracellular calcification of VSMCs which is induced by osteogenic medium through a Notch1-RBP-Jκ-dependent signaling pathway (Figures 1 and 2). Importantly, we proved that OPG reduces the expression of Notch1 and RBP-Jκ as well as its downstream target Msx2 in a dose dependent manner (Figures 3 and 4). These facts suggest that OPG is a negative regulator of Notch1 signaling pathway and the inhibition of osteogenic conversion by OPG occurs through the down regulation of Notch1-RBP-Jκ signaling pathway. Although the mechanisms involved in this regulation of OPG are not fully defined, one of potential explanation is that OPG acts as a decoy receptor for the receptor activator of nuclear factor kappa B ligand (RANKL) in VSMC, by binding RANKL, inhibits NF-κB, subsequently down regulating the expression of Notch1 signaling pathway, thus deteriorating the mineralization in VSMCs caused by the osteogenic stimuli. However, the elucidation of this mechanism will require further confirmation. In addition, whether osteoprotegerin is necessary for the activation of Notch1-RBP-Jκ/Msx2 signaling pathway in VSMC merits additional investigation.

It should also be noted that although our observations in the present study indicate a strong positive association of OPG with the inhibition of the Notch1/RBP-Jκ signaling by showing an excellent agreement with the effect of DAPT, our results also showed that DAPT dramatically reduced but insufficiently abolished the VSMC calcification stimulated by osteogenic medium, and that the reduction of the calcium content is even greater in OPG treated VSMC compared with DAPT treated VSMC whereas the Notch1 levels are comparable between these two groups. These observations imply that multiple signaling pathways may be involved in the effect of OPG on the regulation of VSMC mineralization. For example, our previous study found that OPG could also inhibit calcification of VSMCs via repressing Wnt/β-catenin pathway [49,50]. However, the relationship between these two signaling pathways is unknown so far.

In summary, the present study confirmed that Notch1-RBP-Jκ signaling pathway plays a crucial role in the osteogenic differentiation of VSMCs and may become a new target or direction for prevention and treatment of vascular calcification. We also identified that OPG is a potential inhibitor of VSMC calcification by targeting, at least in part, the notch1-RBP-Jκ dependent signaling pathway resulting the reduction of its downstream gene Msx2. In this regard, our study provides significant novel insight into the molecular mechanism of vascular calcification and also indicates a potential therapeutic target for the vascular diseases. As a secreted glycoprotein, OPG has the operational possibility as a drug that can be used to antagonize media calcification in the future, just like AMGN-0007 (a recombinant osteoprotegerin construct) and to serve as a potential therapeutic agent in the treatment of bone disease [51].
