# Correction: Osteoprotegerin Inhibits Calcification of Vascular Smooth Muscle Cell via Down Regulation of the Notch1-RBP-Jκ/Msx2 Signaling Pathway

**DOI:** 10.1371/annotation/a4dbf2d2-f1f0-4a71-b856-7a54f7914666

**Published:** 2013-08-06

**Authors:** Shaoqiong Zhou, Xin Fang, Huaping Xin, Wei Li, Hongyu Qiu, Siming Guan

The name of the second author was spelled incorrectly. The correct name is Xin Fang. 

